# Rare human mitochondrial HV lineages spread from the Near East and Caucasus during post-LGM and Neolithic expansions

**DOI:** 10.1038/s41598-019-48596-1

**Published:** 2019-10-14

**Authors:** Michel Shamoon-Pour, Mian Li, D. Andrew Merriwether

**Affiliations:** 1 Department of Anthropology, Binghamton University, Binghamton, NY 13902 USA; 2 Department of Biology, Binghamton University, Binghamton, NY 13902 USA

**Keywords:** Population genetics, Biological anthropology

## Abstract

Of particular significance to human population history in Eurasia are the migratory events that connected the Near East to Europe after the Last Glacial Maximum (LGM). Utilizing 315 HV*(xH,V) mitogenomes, including 27 contemporary lineages first reported here, we found the genetic signatures for distinctive movements out of the Near East and South Caucasus both westward into Europe and eastward into South Asia. The parallel phylogeographies of rare, yet widely distributed HV*(xH,V) subclades reveal a connection between the Italian Peninsula and South Caucasus, resulting from at least two (post-LGM, Neolithic) waves of migration. Many of these subclades originated in a population ancestral to contemporary Armenians and Assyrians. One such subclade, HV1b-152, supports a postexilic, northern Mesopotamian origin for the Ashkenazi HV1b2 lineages. In agreement with ancient DNA findings, our phylogenetic analysis of HV12 and HV14, the two exclusively Asian subclades of HV*(xH,V), point to the migration of lineages originating in Iran to South Asia before and during the Neolithic period. With HV12 being one of the oldest HV subclades, our results support an origin of HV haplogroup in the region defined by Western Iran, Mesopotamia, and the South Caucasus, where the highest prevalence of HV has been found.

## Introduction

The major subclade of R0, haplogroup HV has a pivotal position in human mitochondrial (mtDNA) phylogeny as the ancestral clade to haplogroup H-the most common clade in Europe^1^ and the best-defined mtDNA haplogroup according to Phylotree^2^. Comprising the largest number of identified sublineages^2^, including the revised Cambridge Reference Sequence (rCRS)^3^, haplogroup H is one of twelve subclades of HV, the rest of them being: HV0, HV1, HV4, HV-16311C (consisting of HV6-11, HV14-17 and HV22-24), HV-73G (comprising HV2 and HV20), HV5, HV12, HV13, HV18, HV19 and HV21^2^. HV lineages are often divided into three subclades according to their geographic dispersal: the primarily European H and V (a subclade of HV0), and HV*(xH,V), which consists of the rest of the HV lineages. All three are present across Eurasia and North Africa, with HV*(xH,V) lineages being more prevalent in the Near East and the Caucasus^1,4^.

Within Europe, HV*(xH,V) lineages are rare or absent in the north and west, but more common among southern and eastern Europeans. The frequency of HV*(xH,V) peaks in just over 4% in Belarus, Bulgaria and Italy, but reaches exceptionally high frequencies of 7% to 9% in certain localities in Italy^5–7^. It has been suggested that HV4, one of the most common HV*(xH,V) subclades in Europe, originated in Eastern Europe about 14 thousand years ago (kya), and that the major subclade of HV4 has been present in the Franco-Cantabrian region since 5 kya^8^. Using mainly European samples, the largest study of HV*(xH,V) so far^7^ estimated that this clade underwent a major expansion during the Last Glacial Maximum (LGM), and that a glacial refugium origin is likely for many of the southern Italian HV*(xH,V) lineages. Despite the remarkably old age inferred for certain HV*(xH,V) subclades in this region^7^, ancient DNA (aDNA) studies fail to provide evidence for the presence of haplogroup HV*(xH,V) in pre-Neolithic Italy. In fact, haplogroup HV*(xH,V) is not very common among ancient European individuals studied so far, with the oldest case belonging to a 7,000 year-old Linear Pottery Culture (LBK) individual from Central Europe^9^.

**Figure 1:**
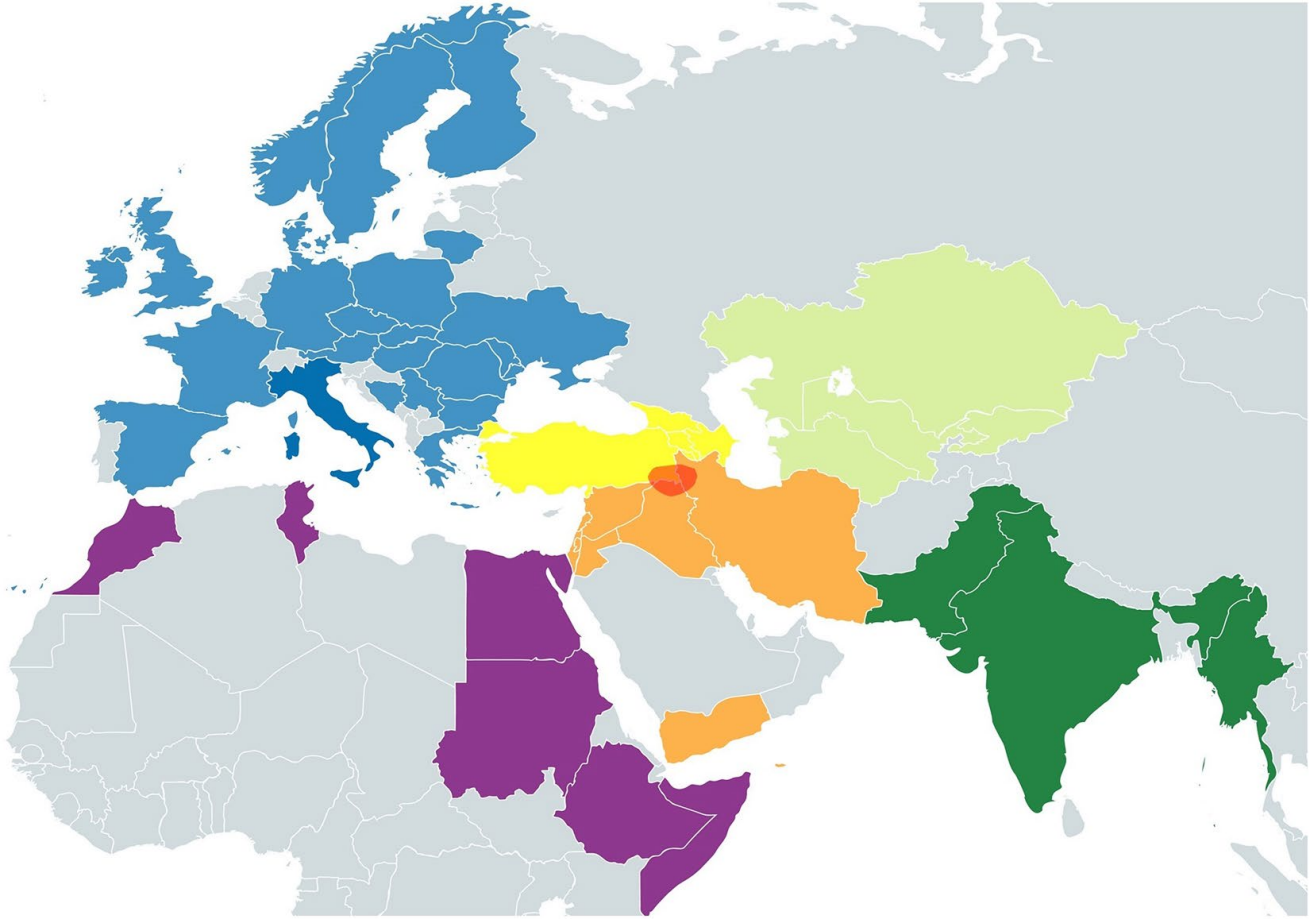
Map of populations included in this study. Colors correspond to six major regions of Africa (purple), Anatolia and South Caucasus (yellow), Central Asia (light green), Europe (blue), Near East (orange), and South Asia (dark green). The approximate boundaries of the Assyrian homeland are demarcated in red.

A reliable estimate for the prevalence of haplogroup HV*(xH,V) across the Near East and the Caucasus proves to be difficult, mainly due to the lack of differentiation between H and HV*(xH,V) in earlier studies that identified subclades solely based on HVS-1 and HVS-2 sequences. HV*(xH,V) reaches its highest frequency and diversity in the region defined by Iranian plateau, Mesopotamia (Iraq), and South Caucasus. The highest frequency of HV*(xH,V) observed in a large-scale study so far is 11% for Iranian Persians^10^. HV*(xH,V) is present at considerably high frequencies also among Iranian Azeris (8.8%) and Qashqais (6.2%)^10^. Similar frequencies (7.1–10.8%) have been reported from Iraq by several studies^11–13^ which represented Arabs as well as Iraqi minority groups, namely Assyrians, Kurds, and Mandaeans. Based on small sample sizes (<30), HV*(xH,V) frequencies of about 7% has been reported from the South Caucasus countries (Armenia, Azerbaijan, and Georgia)^14^. A recent study suggests higher frequencies of HV*(xH,V) among Armenians from the Ararat (11.5%) and Artsakh (8.1%) regions^15^. Reports from Turkey vary greatly (4–24%), but combining 490 samples from five studies puts the average frequency of HV*(xH,V) at 5.1%^14,16–19^. MtDNA data from the ancient Near East is limited, however, aDNA studies have revealed the presence of HV*(xH,V) lineages in Iran (HV), Levant (HV*, HV1b2), southeast Anatolia (HV*, HV8) and South Caucasus (HV*, HV1a, HV12)^15,20–23^. The oldest cases of HV*(xH,V) reported so far are from Tell Halula in Syria (>9 kya)^21^, and Ganj Dareh in Western Iran (8.8 kya)^22^, with the latter individual being the only representative of the ancestral node of HV haplogroup. Previous studies of HV*(xH,V) have emphasized European subclades^10,11^-an approach dictated, to some degree, by a longstanding underrepresentation of non-European lineages. Given the higher prevalence and diversity of haplogroup HV*(xH,V) in the greater Near East, including the Caucasus, this study aims to improve our understanding of the phylogeography of HV*(xH,V) by turning the focus on the subclades present in these regions. To do this we synthesized HV*(xH,V) mitogenomes from recent studies and commercial ancestry tests, with new mitogenomes from the Assyrian population first described here. Samples from this ethno-religious minority are especially relevant because, prior to the genocide and dispersal of 1914–1919^24^, Assyrians resided in a territory (Fig. 1) presently divided between northern Iraq, southeastern Turkey, and northwestern Iran-the three regions with the highest reported frequencies of HV*(xH,V). Today, a majority of Assyrians live in diaspora in the West, and after Iraq, the United States is home to the second largest Population of Assyrians in the world^25^.

## Results and Discussion

Surveying samples from 153 unrelated Assyrian participants of the Assyrian Genetic Project, we identified 27 HV*(xH,V) mitogenomes, amounting to 17.6% of the sample. This is by far the highest frequency of HV*(xH,V) reported for a single population, with the exception of the reports based on small (<30) sample sizes. The new mitogenomes were assigned to five major HV subclades, namely HV1, HV4, HV12, HV-16311, and HV18. We combined Assyrian and previously reported mitogenomes, a total of 315 (Supplementary Table S1), to reconstruct the phylogenetic tree of the HV*(xH,V) subclades (Supplementary Figs S1 and S2). Within HV*(xH,V), we identified 15 new subclades hereby designated as HV1a1a3, HV1a1a4, HV1b4, HV1b5, HV4b1, HV4b2, HV9b1, HV12a1a, HV12b1b, HV15a, HV16a, HV16b, HV18a, HV25 and HV26. Table 1 summarizes the age of the subclades as estimated by two dating techniques (Bayesian and *ρ*-Statistic) and three mtDNA mutation rates (the corrected molecular clock mutation rate^26^, and two mutation rates calibrated with ancient mitogenomes^27,28^).

**Table 1:** Age estimates of HV subclades.

Haplogroup	Age Estimates (kya)											
	Bayesian (BEAST)						*ρ* Statistic					
	Corrected rate		aDNA		aDNA		Corrected rate		aDNA		aDNA	
	(Soares *et al*.^26^)		(Brotherton *et al*.^28^)		(Fu *et al*.^27^)		(Soares *et al*.^26^)		(Brotherton *et al*.^28^)		(Fu *et al*.^27^)	
HV1	19.6	(15.5–24.3)	14.3	(11.4–17.9)	13.1	(10.4–16.0)	23.1	(13.4–33.2)	21.1	(16.8–25.5)	19.0	(15.1–22.9)
HV1a	16.3	(13.4–19.2)	12.1	(9.9–14.2)	11.0	(9.2–12.8)	18.8	(11.4–26.4)	17.4	(14.0–20.7)	15.6	(12.6–18.6)
HV1a1	13.0	(10.3–16.2)	9.7	(7.8–11.6)	8.8	(7.2–10.6)	15.9	(8.7–23.3)	14.8	(11.5–18.2)	13.3	(10.3–16.3)
HV1a1a	11.4	(8.2–14.5)	8.7	(6.8–10.7)	7.9	(6.3–9.7)	15.6	(8.1–23.5)	14.6	(11.1–18.1)	13.1	(10.0–16.3)
HV1a1a1-HV1a1a3	2.9	(1.2–5.1)	2.4	(0.9–3.9)	2.2	(0.9–3.6)	4.2	(0.1–8.3)	4.0	(2.0–6.0)	3.6	(1.8–5.4)
HV1a1a4	6.1	(2.7–9.6)	5.1	(2.3–7.8)	4.8	(2.3–7.3)	7.9	(2.1–13.8)	7.5	(4.8–10.3)	6.8	(4.3–9.3)
HV1a2	12.5	(8.5–16.1)	9.3	(7.0–11.9)	8.5	(6.5–10.7)	8.0	(4.5–11.3)	7.5	(5.9–9.2)	6.8	(5.3–8.2)
HV1a3	8.0	(3.7–12.8)	6.6	(3.2–9.9)	6.2	(3.1–9.0)	6.3	(2.4–10.2)	6.0	(4.2–7.9)	5.4	(3.7–7.1)
HV1b	16.2	(13.3–19.3)	12.0	(9.7–14.2)	11.0	(9.0–12.9)	17.2	(11.3–23.3)	16.0	(13.3–18.7)	14.4	(12.0–16.8)
HV1b + 152 C	13.8	(10.6–16.9)	10.2	(8.0–12.5)	9.3	(7.3–11.3)	13.2	(7.4–19.2)	12.4	(9.7–15.1)	11.2	(8.7–13.6)
HV1b1	8.8	(5.2–13.0)	7.1	(4.3–9.9)	6.7	(4.1–9.0)	10.3	(4.8–15.9)	9.7	(7.1–12.4)	8.8	(6.4–11.1)
HV1b2	4.9	(2.4–8.0)	4.0	(2.1–6.3)	3.8	(2.0–6.0)	2.9	(1.1–4.7)	2.8	(1.9–3.7)	2.5	(1.7–3.3)
HV1b3	10.7	(7.0–14.2)	8.0	(5.5–10.4)	7.3	(5.0–9.4)	16.2	(8.2–24.5)	15.1	(11.4–18.8)	13.6	(10.2–16.9)
HV1b4	2.6	(0.3–5.6)	2.2	(0.3–4.5)	2.1	(0.3–4.3)	1.3	(0–3.8)	1.3	(0–2.6)	1.1	(0–2.2)
HV1b5	7.1	(2.5–11.7)	5.5	(2.0–8.8)	5.1	(2.1–8.1)	6.5	(8–12.5)	6.3	(3.5–9.1)	5.6	(3.1–8.1)
HV4	17.3	(14.4–20.6)	12.5	(10.5–14.6)	11.4	(9.5–13.4)	15.8	(11.4–20.1)	14.7	(12.7–16.7)	13.2	(11.4–15.0)
HV4a	16.1	(13.3–19.3)	11.7	(9.6–13.6)	10.6	(8.6–12.4)	15.2	(9.5–21.2)	14.2	(11.6–16.9)	12.8	(10.4–15.2)
HV4a1	13.2	(10.0–16.3)	9.5	(7.6–11.6)	8.6	(6.8–10.4)	12.1	(7.4–16.9)	11.4	(9.2–13.6)	10.3	(8.3–12.3)
HV4a2	12.5	(8.7–15.8)	9.1	(6.9–12.3)	8.2	(6.2–10.2)	11.6	(4.8–18.7)	11.0	(7.8–14.3)	9.9	(7.0–12.8)
HV4a2a	4.2	(1.7–7.2)	3.5	(1.4–5.8)	3.3	(1.4–5.4)	7.4	(0–15.3)	7.0	(3.3–10.7)	6.3	(3.0–9.6)
HV4a2b	6.7	(2.6–11.0)	5.1	(2.1–8.2)	4.7	(2.2–7.6)	7.9	(1.5–14.5)	7.5	(4.5–10.6)	6.8	(4.0–9.5)
HV4b	13.5	(9.7–17.4)	9.8	(7.3–12.4)	8.9	(6.7–11.3)	15.2	(7.8–22.9)	14.2	(10.8–17.7)	12.8	(9.7–15.9)
HV4b1	3.9	(0.9–7.6)	3.2	(0.7–5.8)	3.0	(0.8–5.4)	3.9	(0–8.4)	3.8	(1.6–6.0)	3.4	(1.4–5.3)
HV4b2	2.7	(0.5–5.4)	2.1	(0.4–4.1)	2.0	(0.4–3.8)	3.9	(0–8.4)	3.8	(1.6–6.0)	3.4	(1.4–5.3)
HV12	16.6	(13.3–20.1)	12.2	(9.8–14.5)	11.1	(8.9–13.3)	19.2	(12.3–26.2)	17.7	(14.6–20.9)	16.0	(13.2–18.7)
HV12a	13.5	(9.7–17.5)	9.9	(7.3–12.7)	9.0	(6.7–11.5)	18.6	(8.1–29.7)	17.2	(12.4–22.1)	15.5	(11.1–19.9)
HV12a1	4.9	(2.1–7.9)	3.8	(1.7–6.1)	3.5	(1.6–5.6)	5.2	(0.4–10.1)	5.0	(2.7–7.4)	4.5	(2.4–6.6)
HV12a1a	2.8	(1.1–4.8)	2.3	(0.8–3.9)	2.1	(0.8–3.6)	1.9	(0–4.1)	1.9	(0.8–3.0)	1.7	(0.7–2.7)
HV12b	13.2	(9.5–16.9)	9.8	(7.6–12.2)	8.8	(6.8–11.0)	14.0	(8.0–20.1)	13.1	(10.4–15.9)	11.8	(9.3–14.3)
HV12b1	—	—	—	—	—	—	12.7	(7.7–17.7)	11.9	(9.6–14.3)	10.7	(8.7–12.8)
HV12b1a	3.8	(1.1–6.7)	2.9	(0.8–4.6)	2.7	(0.9–4.8)	6.5	(0.8–12.5)	6.3	(3.5–9.1)	5.6	(3.1–8.2)
HV12b1b	10.0	(5.4–14.0)	7.7	(4,8–10.1)	7.0	(4.4–9.1)	13.4	(6.5–20.5)	12.6	(9.3–15.8)	11.3	(8.4–14.2)
HV14	12.1	(8.1–15.8)	9.2	(6.6–11.5)	8.4	(6.2–10.6)	13.4	(6.7–20.2)	12.6	(9.5–15.7)	11.3	(8.7–13.9)
HV14a	10.3	(6.4–13.8)	8.0	(5.4–10.2)	7.3	(5.0–9.2)	11.2	(6.3–16.3)	10.7	(8.1–13.2)	9.6	(7.3–11.9)
HV14a1	6.7	(3.5–10.3)	5.4	(2.9–8.0)	5.0	(2.6–7.3)	10.6	(4.1–17.3)	10.1	(7.0–13.1)	9.0	(6.3–11.8)
HV18	10.3	(5.0–15.2)	8.1	(4.2–11.2)	7.6	(4.2–10.1)	7.1	(3.0–11.3)	6.8	(4.8–8.8)	6.1	(4.3–7.9)
HV18a	2.9	(0.7–5.7)	2.4	(0.6–4.6)	2.3	(0.6–4.5)	2.6	(0–5.5)	2.5	(1.1–4.0)	2.3	(1.0–3.6)
HV16311	17.3	(14.7–20.3)	12.7	(10.7–13.8)	11.6	(9.8–13.4)	14.0	(11.8–16.2)	13.1	(10.5–15.2)	11.8	(9.9–13.7)
HV9b1	1.9	(0.3–3.9)	1.7	(0.3–3.3)	1.6	(0.3–3.2)	2.6	(0–6.2)	2.5	(0.7–4.3)	2.3	(0.7–3.9)
HV15a	3.9	(1.1–7.2)	3.2	(0.9–5.9)	3.0	(0.7–5.3)	5.2	(0–10.8)	5.0	(2.4–7.6)	4.5	(2.1–6.9)
HV16a	5.3	(2.1–8.9)	4.5	(1.7–7.5)	4.3	(1.8–7.0)	7.0	(2.1–12.0)	6.7	(4.3–9.1)	6.0	(3.9–8.1)
HV16b	1.9	(0.2–4.1)	1.7	(0.1–3.5)	1.6	(0.1–3.4)	1.3	(0–3.8)	1.3	(0–2.6)	1.1	(0–2.2)
HV25	1.6	(0.0–4.2)	1.4	(0.1–3.5)	1.3	(0.0–3.2)	1.3	(0–3.8)	1.3	(0–2.6)	1.1	(0–2.2)
HV26	1.6	(0.0–3.8)	1.3	(0.0–3.0)	1.2	(0.0–2.8)	1.3	(0–3.8)	1.3	(0–2.6)	1.1	(0–2.2)

Values in parentheses state the age ranges as 95% highest posterior density or confidence intervals, for Bayesian and *ρ*-Statistic estimates, respectively.

Across the HV*(xH,V) tree, two distinct patterns are recognizable:

1. In several subhaplogroups, Italian lineages coalesce with the Near East and the Caucasus lineages at the bases of the clades. This pattern is strikingly similar across several pre-Neolithic and Neolithic subclades (HV1a1, HV1a2, HV1b3, HV4a2, HV4b, HV18).
2. Certain HV*(xH,V) subclades are completely absent in Europe. Theses subclades are primarily composed of lineages found in the Near East, the Caucasus and South Asia. The coalescent times of most of these subclades point at their pre-Neolithic (HV12, HV14) and early Neolithic (HV1a3, HV1b1) origins. This category also includes subclades first described in this study (HV1b5, HV12a1a, HV12b1b).

We focus our analysis on these two groups of subclades, as they provide insight into the earliest Europe-Near East and Near East-South Asia branching events within the HV*(xH,V) clade, as well as the time and place of origin of the oldest non-European HV*(xH,V) subclades.

**Figure 2:**
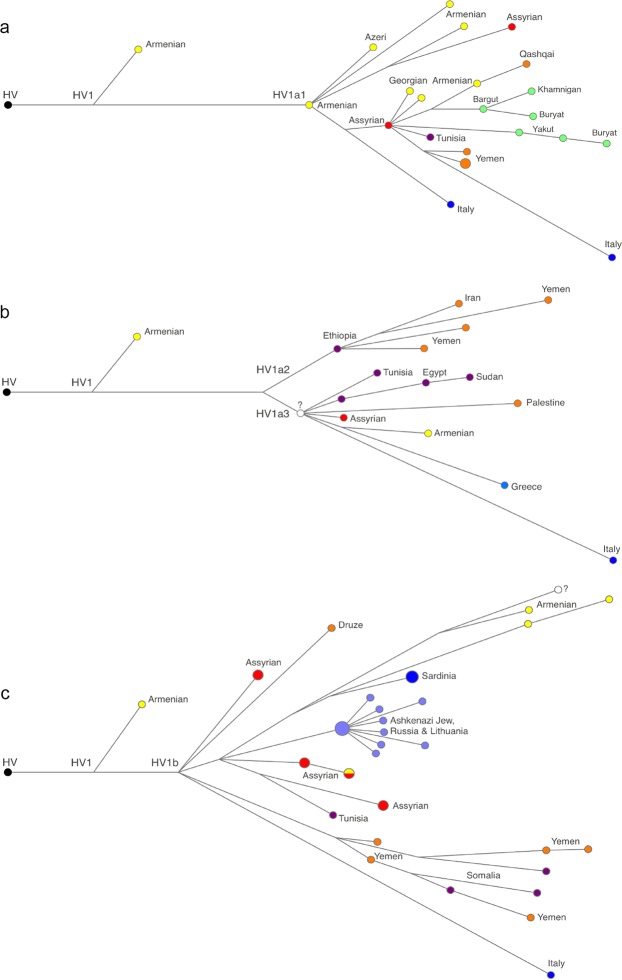
Median-Joining Network of haplogroup HV1. Networks of the three major HV1 subclades of are displayed separately. Each network comprise all available mitogenomes for HV1a1 (**a**), HV1a2-HV1a2 (**b**) and HV1b (**c**) subclades, including the Assyrian mitogenomes first described in this study (red).

HV1 is one of the most prevalent and most diverse subclades of HV*(xH,V). Previous studies have estimated the age of HV1 at between 17 and 28 kya^7,26,29^, making it one of the oldest subclades of HV. A vast majority of the HV1 lineages belong to HV1a and HV1b subclades, dated similarly at 17 kya^7,29^. Using 40 mitogenomes representing more populations than previous studies, we estimate the age of HV1a at 16.3–18.8 kya (corrected mutation rate) and 11.0–17.4 kya (aDNA-calibrated rates). Of the three HV1a branches, HV1a1 is the oldest. According to the median-joining network analysis, an Armenian mitogenome represents the ancestral node of HV1a1 (Fig. 2). The vast majority of lineages in this subclade belong to the HV1a1a subclade, the exception being four mitogenomes representative of Armenians, Assyrians and Azeris. The ancestral node of HV1a1a is also represented by an Assyrian mitogenome. Notably, an Italian lineage branches off the root of HV1a1a, representing a schism between the ancient Italian HV*(xH,V) lineages, and the rest of lineages descending from the Assyrian mitogenome. Our Bayesian estimation puts the age of this branch at 11.4–15.6 kya.

Within haplogroup HV*(xH,V), HV1a1a is unusual for its extraordinary geographic distribution. Although rare, HV1a1a is present in the Caucasus, the Near East, Central and Northeast Asia, North Africa, and Italy. A previous study designating Siberian-specific HV1a1a1 and HV1a1a2 lineages has speculated about the West Asian origin of these subclades^30^. We were able to identify a sister subclade of HV1a1a1, hereby designated as HV1a1a3, comprising Iranian and Armenian lineages. Sharing the deletion at position 249, the HV1a1a1-HV1a1a3 clade (Supplementary Fig. S1) has an estimated age of 2.9–4.2 kya. We can speculate that the Mongolian/Siberian HV1a1a1 subclade is the result of migrations from Mesopotamia and Iran to Central Asia during the expansion of the Persian Achamenid Empire 2.5 kya^31^. Within HV1a1a, we also introduce an older subclade, HV1a1a4, which represents shared ancestry of its Italian and Yemeni lineages at 6.1–7.9 kya (Supplementary Fig. S1).

The HV1a2 and HV1a3 subclades are distinctive from HV1a1 by virtue of their predominant presence in Africa and Yemen. The phylogeny of the HV1a2, the larger and older of the two (6.8–12.5 kya), shows some parallels with that of HV1a1: Assyrian, Armenian, and Italian mitogenomes are present, with the latter comprising a deep branch that separates it from the root with eight mutations (Fig. 2).

According to Phylotree Build 17^2^, HV1b contains two main branches: HV1b1, found in Arabia and East Africa, and a larger subclade defined by the T152C! mutation. HV1b-152 is subsequently divided into HV1b2 and HV1b3, present in Eastern Europe and Armenia, respectively. Our Assyrian samples informed the phylogeny of HV1b subclade by revealing four new haplotypes (Fig. 2). Within HV1b-152, we were able to identify two new branches: HV1b4 (defined by mutations T4047C and C10095T) and HV1b5 (defined by C16234T) (Supplementary Fig. S1). With regard to the relationship between Italian and Near Eastern lineages, the phylogeny of HV1b resembles that of previously described HV1a subclades; an Italian lineage forms an extremely deep branch off the root of HV1b (12.0–17.2 kya), and several Assyrian and Armenian mitogenomes form the most ancestral nodes. Curiously, three Sardinian mitogenomes^32^ represent a younger branch (8 kya) within the otherwise exclusively Armenian HV1b3 subclade.

Unlike HV1, HV4 is a primarily European clade, with its highest frequencies reported in Eastern Europe. Within HV4, however, HV4a2 and HV4b are known for their different geographic distributions (Supplementary Fig. S3). Unlike the exclusively European HV4a1, HV4a2 has been found among Assyrians and Armenians, as well as in Jordan, Egypt and Italy. Here we doubled the number of full mtDNA sequences representing HV4a2 by adding seven new Assyrian mitogenomes. We estimate the age of HV4a2 and the exclusively Assyrian HV4a2a subclades at 8.2–12.5 and 3.3–4.2 kya, respectively. We also identified an Assyrian branch within HV4b, a small subclade previously reported from the Caucasus (Supplementary Fig. S1).

**Figure 3:**
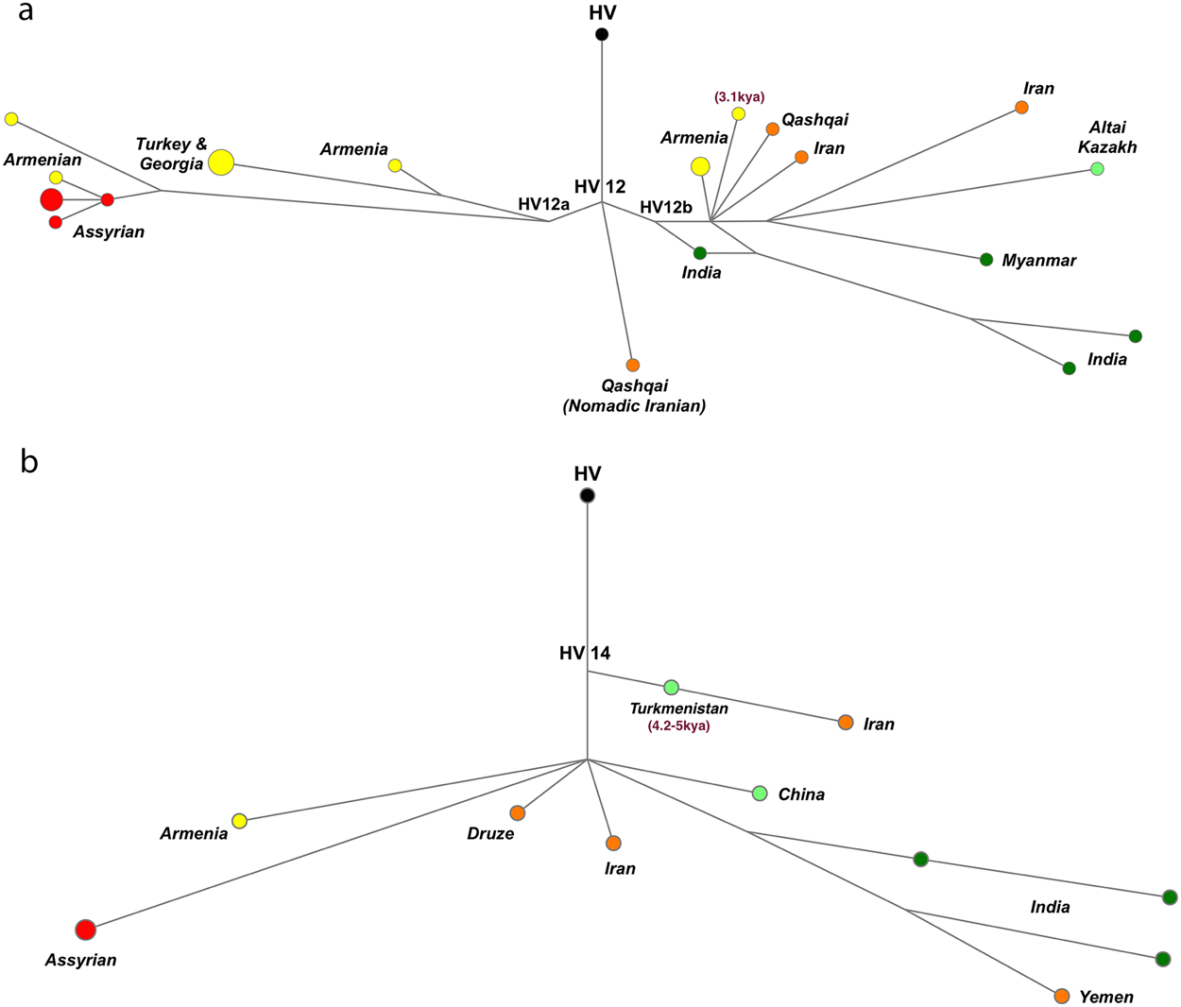
Median-Joining Network of non-European HV haplogroups. Similar phylogeographies of HV12 (**a**) and HV14 (**b**) reveal their pre-Neolithic origin in Iran, with deep branches expanding westward into Mesopotamia and South Caucasus, and eastward into South Asia.

With their territory stretching from the South Caucasus to South Asia, the phylogeography of HV12 and HV14 haplogroups reveal a distinctive pattern among the HV*(xH,V) subclades. Given the ages of these subclades, HV12 and HV14 provide important information about the origin and early expansion of HV*(xH,V). Particularly concerning HV12, previous studies have estimated its origin at 19.4 to 21.3 kya, a short time after the coalescence of the entire HV haplogroup around 21.9 to 28 kya^7,29^. Using a larger number of sequences, including five new Assyrian mitogenomes, we estimate the age of HV12 at a similar 19.2 kya (*ρ* statistics, corrected mutation rate), although Bayesian analysis suggests a younger (16.6 kya) age. The median-joining network (Fig. 3) illustrates a pattern of geographic separation between the two main branches of HV12. HV12a, the older subclade, is exclusive to South Caucasus and Anatolia. HV12b is the younger branch (8.8–14.0 kya) and has been found in Iran, India, and sporadically as far as Central and Southeast Asia. A lineage branching off the root of HV12 consists of a single Qashqai mitogenome. Nomadic pastoralists of southern Iran, Qashqais are the Turkic-speaking people who previously resided in the Iranian section of the South Caucasus^33^.

A rare haplogroup, HV14 has been previously reported from Iran, India, Sri Lanka and Yemen, as well as one individual from ancient Turkmenistan (4.5 kya)^22^. A subclade of this haplogroup (HV14a1) is surprisingly prevalent (5%) in the southernmost states of India as well as Sri Lanka, with its age estimated at 11 kya^34^. Here we add three new mitogenomes (two Assyrians, one Druze from the FamilyTreeDNA database) to the existing pool of only eight HV14 mitogenomes. We estimate the age of HV14 haplogroup at 12.1–13.4 kya (corrected mutation rate) or 8.4–12.6 kya (aDNA-calibrated rates). Although the small number of available mitogenomes makes any conclusive interpretation difficult, the phylogeography of HV14 nevertheless suggests southern Iran as a likely point of origin for this haplogroup. Moreover, two highly differentiated HV14 mitogenomes reported from a province in southern Iran (Kerman) suggest an old age for HV14 in this region. Additionally, one of the Iranian mitogenomes and the ancient Turkmenistan individual form together a “Pre-HV14” subclade (Supplementary Fig. S2) that had reached Central Asia 4.5 kya^22^.

**Figure 4:**
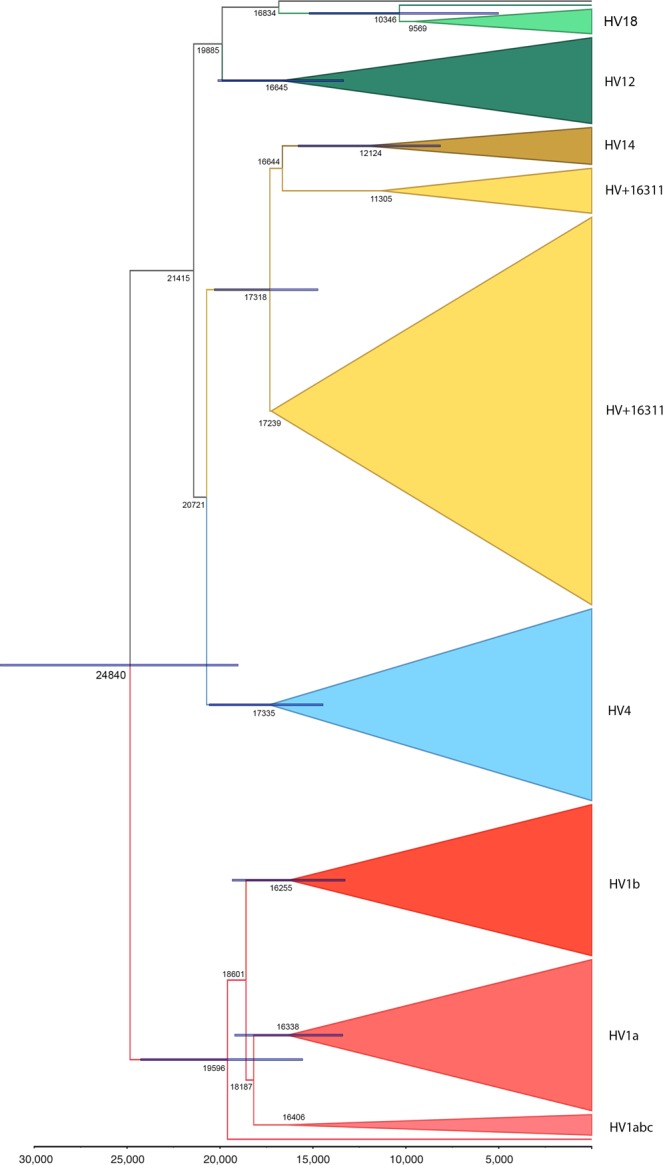
Bayesian phylogeny of the 315 HV*(xH,V) mitogenomes. Values represent the estimated age of each subclade according to the corrected mutation rate^26^, with blue bars depicting the 95% HPD intervals. Similar to the results of *ρ*-Statistic analysis (Supplementary Figs S1 and S2), all major subclades express pre-Neolithic origins, with HV18 being the exception.

The Near Eastern and South Asian dispersion of HV14 is particularly curious, given that it is a subclade of the otherwise European HV-16311. Furthermore, one ancestral lineage of HV14 in fact lacks the haplogroup-defining T16311C mutation. With T16311C being highly recurrent, we examined the alternative phylogeny of HV14 as a clade independent from HV-16311. Utilizing all available HV-16311 mitogenomes, a total of 135, our analysis did not provide evidence against the current position of HV14 as a subclade of HV-16311 (Supplementary Fig. S2, Fig. 4). Given the phylogeography of HV-16311 and the age estimates, it is likely that HV-16311 first emerged in Anatolia around 17 kya. While most of HV-16311 lineages soon expanded westward into Europe, probably during the post-LGM and Neolithic migrations, an eastward migration introduced the HV14 or its precursors to Iranian plateau by 12 kya.

Revisiting the phylogeography of HV*(xH,V) in the light of the aDNA, archaeological, and paleoclimatological discoveries, our findings are as follows:

### HV*(xH,V) subclades shared between Italy and the Near East characterize two distinct migratory events

- **Post-Glacial migration from the Near East to Europe:** Parallel phylogenies of HV1a1, HV1a2, HV1b and HV4b point to a division of exclusively Italian lineages from the Caucasus/Near East lineages no later than 12 kya. The phylogeography of these subclades (Supplementary Fig. S1) suggests an expansion from the South Caucasus or northern Mesopotamia into Europe, as well as to Africa and Asia. Our proposed ages of 12–16 kya for branching of the oldest European HV*(xH,V) lineages from the Caucasus/Near East lineages correlate with the start of the first post-LGM warm period in Europe around 14.7 kya^35^. Studies of other mtDNA haplogroups have provided substantial evidence for a migratory event of similar age being responsible for introducing a variety of Near Eastern haplogroups (I, W, J-T) to Europe^36,37^. The aDNA evidence also reveals a major change in the mitochondrial makeup of Europe around 14.5 kya^38^, although to date HV*(xH,V) has not been found in pre-Neolithic Europe or Near Eastern sites. We attribute the lack of aDNA evidence to the small population size of HV*(xH,V) clade before the Neolithic period (Supplementary Fig. S4), and predict that further study of pre-Neolithic sites in Southern Europe, especially Italy, will reveal the presence of HV*(xH,V) lineages, likely from HV1a, HV1b and HV4b subclades. Given the significance of South Caucasus in phylogeography of HV*(xH,V), it is noteworthy that genomic similarities have been observed between the Caucasus “Satsurblia” individuals (10–13 kya) and the “Villabruna Cluster” Europeans (7–14 kya)^39^. It should be noted that these results do not necessarily indicate a relationship between these two groups, and data can also be explained by population structure.
- **Neolithic expansion into Europe:** Several HV*(xH,V) subclades, including HV1b3 and HV18, point at an early Neolithic connection between Europe and the Near East. More specifically, these subclades represent branching events at 7–11 kya between Italian and South Caucasus lineages (Supplementary Fig. S1). Ancient DNA studies have demonstrated that the first European farmers were descended from Neolithic northwestern Anatolians and Aegeans^40–42^, and that the Neolithic Western Iranians are not ancestral to Neolithic Europeans^43,44^. That said, the possible role of intermediate southeastern and central Anatolian populations in Neolithic migration to Europe has not been ruled out^44,45^ and a recent study has revealed a secondary source of ancestry for Neolithic Europeans^42^. With its Caucasus Hunter Gatherer (CHG)-rich ancestry, this source population is likely related to Neolithic Central or eastern Anatolia. Given the heavy presence of closely related Armenian and Assyrian lineages near the root of these HV*(xH,V) subclades, it is plausible that these lineages were introduced to Europe by Neolithic populations from southeastern or central Anatolia that are yet to be represented in aDNA studies.
- **Expansion into Africa:** Certain HV*(xH,V) subclades seem to have expanded from the Near East and Europe into North Africa starting during the Neolithic period. The oldest of these African HV*(xH,V) subclades are similarly dated at 6.2–8.0 kya (HV1a3) and 6.7–8.8 kya (HV1b1), and are now found across Red Sea in Ethiopia and Somalia. For three reasons, these subclades are most likely of Near Eastern origin: The Yemeni HV1b1 haplotypes are ancestral to the African haplotypes, both HV1a3 and HV1b1 are more common in Yemen than among African populations, and that both subclades branch off the older clades believed to have originated in the Near East and the Caucasus. African and Yemeni haplotypes are also present in two younger subclades, HV4a2b (4.7–6.7 kya) and HV1a1a4 (4.8–6.1 kya), each comprising an Italian lineage. These subclades are likely representative of the early maritime activities in ancient Mediterranean world.

### The non-European HV*(xH,V) subclades point at a pre-Neolithic migration from Iranian plateau to South Asia

Substantial archaeological evidence connects the Indian subcontinent’s earliest Neolithic sites to Mesopotamia and Iran^46^. Genetic studies have suggested that West Eurasian haplogroups were introduced to South Asia from the Near East with a combination of the LGM, early Neolithic and Bronze Age migratory events^34,47^. Recent aDNA findings provide evidence of eastward migration of people from Zagros Mountains to South Asia at least 9 kya^44^. Within HV*(xH,V), similar phylogenies of HV12 and HV14 point at such migratory events. In both haplogroups, the oldest nodes belong to Iranian and Caucasus mitogenomes, and each haplogroup comprises two major branches (Fig. 3). The eastern branches of these haplogroups, HV12b and HV14a1, split from their Near Eastern bases 8.8–14.0 and 5.0–10.6 kya, respectively. Comprising very deep branches of Indian and Iranian lineages, HV12b is unique among HV*(xH,V) subclades in characterizing a pre- or early Neolithic expansion of this haplogroup eastward of Iranian plateau. We also identified a new branch within HV12b; originated 10–13.4 kya, HV12b1b connects Iran to Central (Altai Kazakh) and Southeast Asia (Myanmar). In case of HV14, due to the prevalence of HV14a1 among Dravidian-speaking populations of southern India and Sri Lanka, it has been suggested that this subclade represents the migration of proto-Dravidian people from the Near East to South Asia around 10 kya^34^. That said, the origin of Dravidian languages and the possibility of a Near Eastern connection is highly debated^48^. Nevertheless, the phylogeography of HV14 points to an origin in Iran, and the age of HV14a1 in India suggest its presence in South Asia prior to the Bronze Age expansion of Indo-Aryan languages.

### HV1b2 is an Ashkenazi Jewish subclade with links to Northern Mesopotamia

The new lineages introduced in this study have implications on the possible origin of HV1b2, a star-like subclade of HV1b-152 that is mostly comprised of Ashkenazi Jewish lineages^49^ (Fig. 2). Our updated phylogeography of HV1b-152 suggests its pre-Neolithic origin in South Caucasus or northern Mesopotamia, with HV1b2 positioned close to two Assyrian branches. Given our age estimates for HV1b2, it is conceivable that this clade originated among the displaced Jewish communities during Assyrian and Babylonian captivities (2.5–2.7 kya), and likely remained exclusive to the Jewish populations that settled in Upper Mesopotamia, including Adiabene and Osroene Kingdoms, up to 1.7 kya^50^. Perhaps a similar course of events has resulted in 58% of Georgian Jews belonging to a single haplotype of HV1a1a^51^, a subclade also known to be present among Assyrians, Armenians and Georgians.

Being phylogenetically independent from nuclear DNA, mtDNA is particularly valuable when studying complex demographic events, such as sexually biased migrations and unilateral population structuring^52,53^. A growing number of mitogenomic studies prove the effectiveness of this approach, especially when analysis focuses on a specific subclade^54–57^. Given the underrepresentation of most Near Eastern populations in genomic studies, the relative wealth of full mitogenome data provides a great opportunity to study the complex and long history of human dispersal in Near East. In case of HV*(xH,V), although relatively uncommon, this haplogroup is highly informative to study of prehistoric migrations that connected the Near East to Europe and South Asia. The parallel phylogenies of several HV*(xH,V) subclades reveal a connection between the Italian Peninsula and the South Caucasus populations, likely resulting from at least two (post-LGM, Neolithic) waves of migrations. These findings add to aDNA evidence suggesting a secondary, CHG-rich source of ancestry for Neolithic Europeans. Similarly, the eastern subclades of HV*(xH,V) provide insight into the ancient migratory events between Near East and South Asia.

While the accumulation of full mitogenomes in the past decade has largely advanced our understanding of the phylogeography of major haplogroups, the bulk of mitogenomes comes from studies of European populations, both contemporary and ancient. Given the abundance of novel mtDNA lineages discovered by every study of Near Eastern populations, further research in this region is essential to better understanding of the origin and dispersal of West Eurasian mtDNA clades, and of important demographic events that have shaped the genetic structure of West Eurasian populations.

## Materials and Methods

Participants were selected from Assyrian residents of the United States who could trace their maternal lineages back for at least three generations, and were able to identify their ancestral town or village. All participants provided their written informed consent as per the consent form approved by Binghamton University’s Human Subjects Research Review Committee (protocol number: 3000–13). All methods were performed in accordance with the Binghamton University’s Human Subjects Research Review Committee. Buccal samples were collected from the volunteers using Oragene OGR-500 Saliva Collection Kit (DNA Genotek, Canada) and DNA was extracted according to the manufacturer’ instructions. Prior to high-throughput sequencing, mtDNA content of samples was enriched using a long-PCR method with a novel set of primers that amplified mtDNA in two segments of 8,476 bp (primers 2508F-CATCACCTCTAGCATCACCAGT and 10983R-AGGGGTAGGAGTCAGGTAGTT) and 8,460 bp (primers 10837F-CACAACCACCCACAGCCTAAT and 2727R-GGTCTTCTCGTCTTGCTGTGT). These primers were designed with high specificity, in order to avoid amplification of nuclear DNA, particularly mtDNA pseudo-genes. The two amplicons targeted by these primers also have a smaller overlap compared with previous mtDNA long-PCR methods^14,58,59^ in order to improve evenly distributed coverage during sequencing. The optimized conditions of two separate reactions were as following: TaKaRa (Clontech) LA PCR buffer II with 25 mM Mg2+ (5 *μ*L), TaKaRa 10 mM dNTP mixture (8 *μ*L), TaKaRa LA Taq polymerase (2.5 units), primers (1.25 *μ*L of each), and dH20 to reach a final volume of 50 microliters.

Libraries were prepared using Nextera® XT kits (Illumina, Inc.) and sequencing was carried out on Illumina’s MiSeq benchtop sequencer using the v2 500-cycle reagent kit. Trim Galore v0.4.0 software (Babraham Informatics) was used for quality filtering (PHRED >30) of reads and trimming of adapters. The paired-end reads were mapped using BWA MEM^60^. Further processing of SAM files was carried out utilizing Samtools^61^ along with bcftools and vcfutils^62^. Haplotypes were manually verified using Integrative Genomics Viewer (IGV) v2.3.72^63^. Full mitogenome sequences were aligned using MAFFT v7^64^. Mutations were defined relative to the rCRS, and haplogroups were assigned following PhyloTree Build 17^2^. Network 5.0.0.1 program (Fluxus Technology Ltd, fluxus-engineering.com) was used to construct Median-joining networks^65^. Two different approaches were taken to estimating the coalescence times: (*ρ*) statistic^66^, and the Bayesian MCMC as implemented in Program BEAST2^67^. For both approaches, times were calculated based on the corrected molecular clock mutation rate^26^, as well as mutation rates calibrated with ancient mitochondrial genomes^27,28^. For Bayesian analysis, jModelTest v2.1^68^ determined the Hasegawa Kishino-Yano (HKY)^69^ as the best substitution model for the sample. The Strict molecular clock was used. Three independent runs of 50 million iterations (with the first 5 million discarded as burn-in) were performed and the logs were combined using the LogCombiner v2.4.2. The effective population size was calculated using Tracer v1.6.0, assuming a generation time of 25 years. Tracer was also utilized to visualize the Bayesian Skyline Plots, and FigTree v1.4.2 was used to edit and visualize the phylogenetic tree.

## Supplementary information

Supplementary Table S1

Supplementary Figure S1

Supplementary Figure S2

Supplementary Information

## Data Availability

Complete mitogenome sequences generated in this study are available at the NCBI (GenBank accession numbers MK217113-MK217139).
