# Supplementary material for: Rare human mitochondrial HV lineages spread from the Near East and Caucasus during post-LGM and Neolithic expansions
Source: Sci Rep. 2019 Oct 14;9:14751. doi: 10.1038/s41598-019-48596-1 (PMC6791841; doi:10.1038/s41598-019-48596-1)
Supplement: Supplementary file 4 — Supplementary Information [file 41598_2019_48596_MOESM4_ESM.pdf]

Supplementary Information

Rare human mitochondrial HV lineages spread from the Near East and Caucasus during post-LGM and Neolithic expansions

Michel Shamoon-Pour[1,*], Mian Li[2], and D. Andrew Merriwether[1]

1 Department of Anthropology, Binghamton University, Binghamton, NY, 13902, USA

2 Department of Biology, Binghamton University, Binghamton, NY, 13902, USA

* Correspondence and requests for materials should be addressed to M.S-P., mshamoon@binghamton.edu

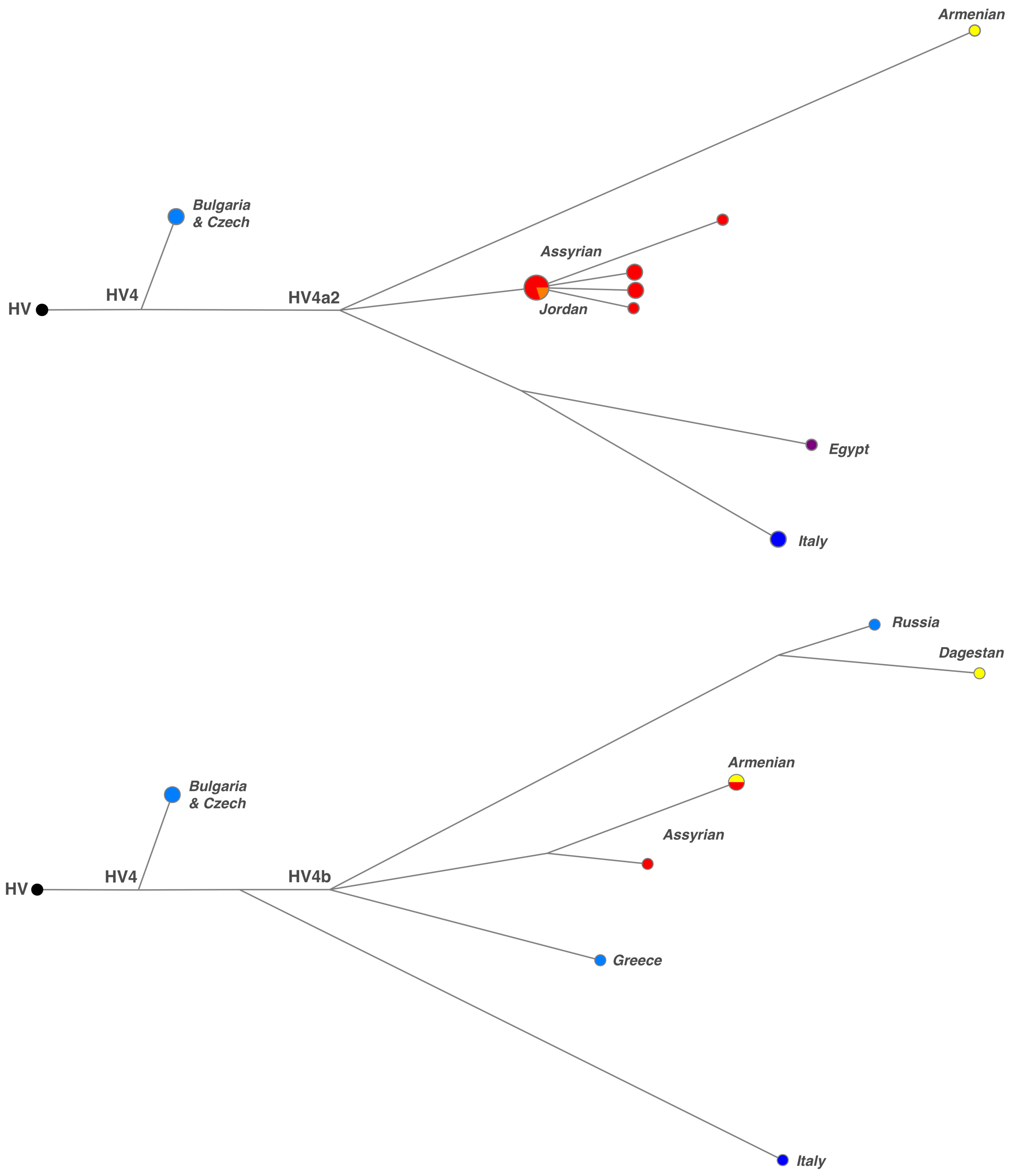

Supplementary Figure S3. Median-Joining Networks of HV4a2 and HV4b. Networks represent all available mitogenomes for each subclade, including the new Assyrian mitogenomes from this study (red).

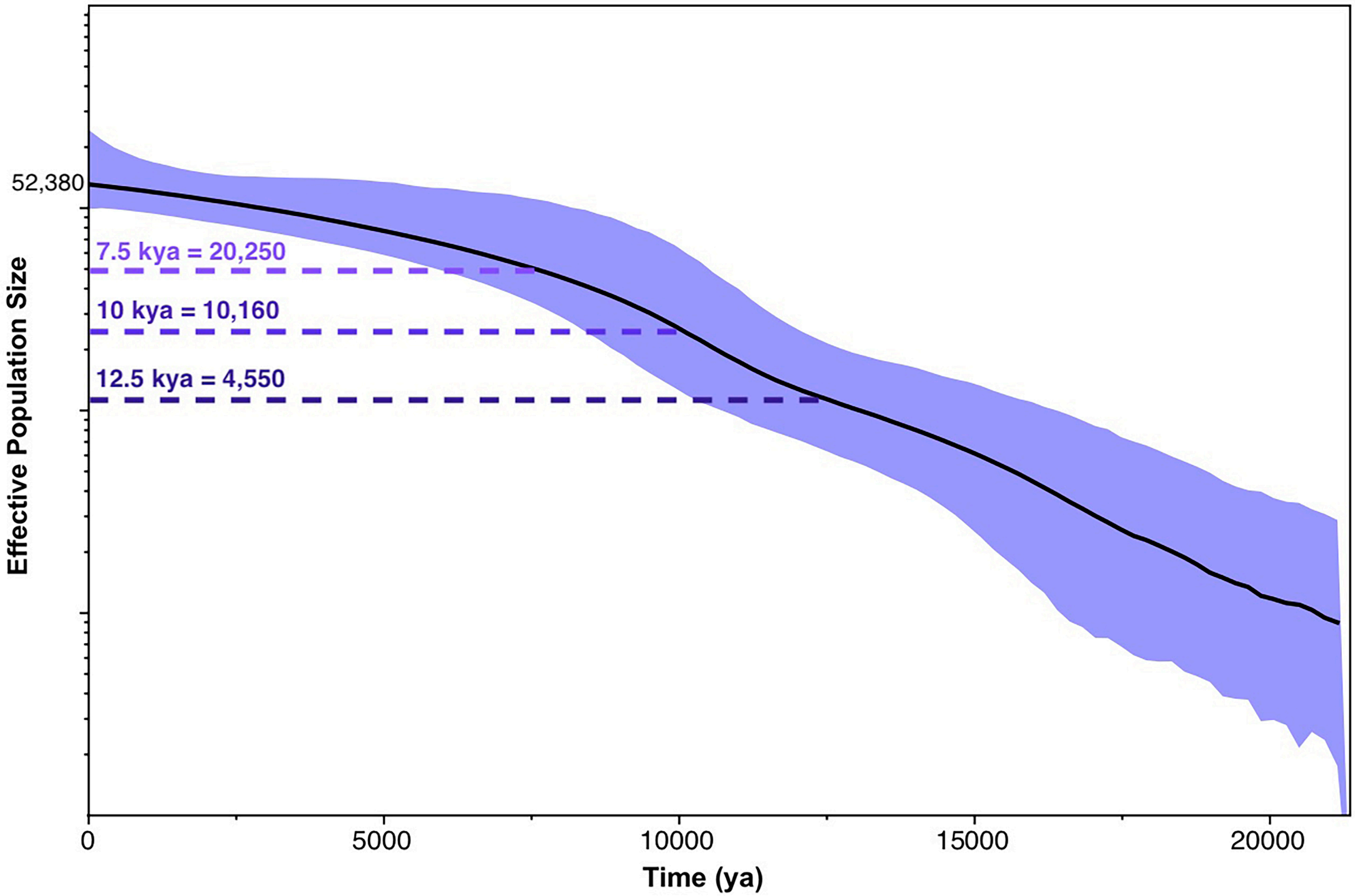

Supplementary Figure S4. Bayesian Skyline Plot of HV*(xH,V) Haplogroup. Effective Population sizes were estimated based on an average generation time of 25 years.
